# Individual COVID‐19 disease trajectories revealed by plasma proteomics

**DOI:** 10.15252/emmm.202114532

**Published:** 2021-07-14

**Authors:** Danish Memon, Inigo Barrio‐Hernandez, Pedro Beltrao

**Affiliations:** ^1^ European Bioinformatics Institute (EMBL‐EBI) Cambridge UK

**Keywords:** Microbiology, Virology & Host Pathogen Interaction, Proteomics

## Abstract

Since the start of 2020, the world has been upended by the pandemic caused by the severe acute respiratory coronavirus type 2 (SARS‐CoV‐2), the causative agent of coronavirus disease 2019 (COVID‐19). It has not only led to a tragic loss of life and terrible economic costs but has also been met with an unprecedented response of the scientific and medical communities. In an effort to better understand this viral infection, scientists around the world generated the largest surge in research in documented history for any topic (Lever & Altman, 2021). A part of this work has included the need to better understand the impact of the virus on human proteins—the key machinery of the cell—and human physiology. In their recent study, Geyer and colleagues (Geyer *et al*, 2021) analyzed a total of 720 proteomes from longitudinal serum samples of 31 hospitalized COVID‐19 patients and control individuals with COVID‐19‐like symptoms but not infected with SARS‐CoV‐2, providing a comprehensive characterization of the plasma proteome changes along the time course of infection.

## Graphical abstract

D. Memon, I. Barrio‐Hernandez, and P. Beltrao discuss the study by Geyer *et al* (in this issue of *EMBO Mol Med*) that analyzes the plasma proteome changes for a longitudinal cohort of COVID‐19 patients.

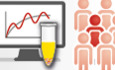

Mass spectrometry (MS)‐based proteomics is an ideal technology to detect and measure the changes in proteins occurring due to the infection. In the context of cell‐based models, this technology has been applied to study how SARS‐CoV‐2 takes control of its target cell in order to identify potential host targeting drugs that could inhibit viral replication (Bouhaddou *et al*, 2020; Gordon *et al*, 2020; Selkrig *et al*, 2021). However, to understand the process of infection and its impact on human physiology, direct measurements of patient material are needed. Plasma‐based proteomics holds the promise of being a quick, non‐invasive assay of the health status of the human body. Proteins in the blood could originate from different parts of the body and contain many relevant disease markers that are already used routinely for clinical decisions. While there are many challenges in analyzing blood samples, MS‐based proteomics is now being run at a larger scale and is already providing fascinating insights into human genetics and disease (Suhre *et al*, 2021).

Plasma‐based proteomics has been previously applied to study the protein differences between healthy and COVID‐19 patients of different severity levels (Messner *et al*, 2020; Shen *et al*, 2020; Shu *et al*, 2020). These studies have identified specific changes in protein abundance for COVID‐19 patients that could be used as predictive markers of disease and potentially also which patients will have the worst prognosis. However, these studies were based on a single time point measurement. In this issue of *EMBO Molecular Medicine*, Geyer and colleagues report on the study of plasma proteome changes for a longitudinal cohort of 31 COVID‐19 patients with an average of 14 samples per patient, during an average period of 31 days (Fig 1A) (2021). Around 300 proteins per sample were quantified in a total of 720 samples, including controls. Similar to the previous studies, they could identify characteristic protein changes occurring in COVID‐19 patients. These include the increased abundance of innate immune proteins and protease inhibitors and decreased abundance of coagulation and lipid homeostasis proteins. Several of these biological processes and specific proteins were also found in previous single time point studies showcasing the reproducibility of the findings across cohorts. However, by using the longitudinal samples, they demonstrate how the contrast between controls and disease can depend strongly on the disease progression. When the patients are sampled at the highest levels of SARS‐CoV‐2 antibodies, instead of the first day of sampling, the specific regulated proteins detected can be substantially different.

**Figure 1:**
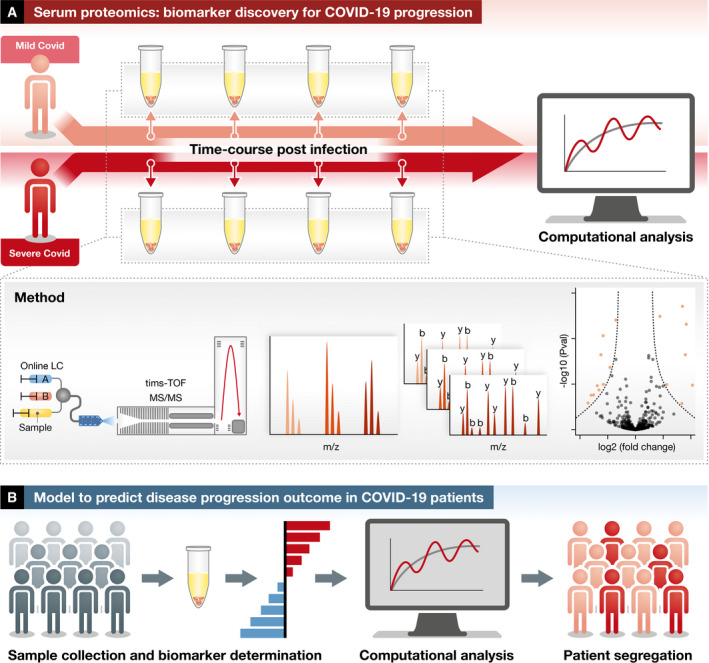
Predictive models of COVID‐19 disease from plasma proteome measurements (A) In this study, blood samples collected from COVID‐19 patients along a time course of infection were used to identify protein abundance levels via MS‐based proteomics. The protein abundance changes can specifically identify patients from controls and have the potential to predict the progression of the disease for the newly diagnosed patients. (B) This study and others argue that routine plasma proteomics could be used to monitor biomarker protein levels in the clinic with computational models used to predict disease progression in COVID‐19 patients.

In addition to comparing COVID‐19 patients with controls, the authors studied how the plasma proteome changes along the time course of infection. This revealed broad trends of decreased abundance of innate immune‐related proteins; increased abundance of lipid homeostasis proteins and coagulation factors; and a more complex dynamical pattern of increased followed by decreased coagulation‐related proteins. When comparing 25 patients that survived with 6 that did not, they identified a small number of proteins that may potentially serve as biomarkers of disease severity, including the pro‐inflammatory acute phase protein ITIH4 and coagulation‐related proteins such as heparin cofactor 2 (SERPIND1), plasma kallikrein, (KLKB1), and plasminogen (PLG). The markers related to inflammation and coagulation were also independently identified as predictive of patient survival in an independent longitudinal plasma proteomic study of COVID‐19 patients (Demichev *et al*, 2021).

This study demonstrates how plasma MS‐based proteomics can be used to study relevant aspects of disease progression and to identify relevant biomarkers of disease and possibly also disease severity. Together with previous studies, this application to COVID‐19 further reinforces the importance of using these technologies as a tool for routine health assessment supplementing the blood tests already used. The findings from the longitudinal study argue for the importance and perhaps even the necessity of monitoring the plasma proteome over multiple time points during disease progression. The availability of plasma proteomics across multiple cohorts could be used in the future for meta‐analysis, which could identify the most robust patterns of protein changes and biomarkers.

This study raises questions related to the future implementation of these approaches, including the use of machine learning in a clinical setting, cross‐platform compatibility, and data access (Fig 1B). As this study highlights, machine learning is critical for taking advantage of large‐scale datasets of patient data, but the implementation and routine use of machine learning models in clinical decisions remains challenging. Similarly, the robustness of biomarkers and predictive models established will need to be accessed across different technological platforms and disease cohorts. For this purpose, it is also critical that these patient data be fully accessible for research purposes while also respecting the privacy concerns of individuals. Proteomic data sharing via controlled access needs to be implemented (Bandeira *et al*, 2021), as it already happens for other biomolecular measurements from patients. Despite these issues, the arguments for routine implementation of plasma proteomics in the clinic are highly strengthened by this work.
